# Antioxidant and Anti-inflammatory Extracts From Sea Cucumbers and Tunicates Induce a Pro-osteogenic Effect in Zebrafish Larvae

**DOI:** 10.3389/fnut.2022.888360

**Published:** 2022-05-09

**Authors:** Alessio Carletti, Carlos Cardoso, Jorge Lobo-Arteaga, Sabrina Sales, Diana Juliao, Inês Ferreira, Paula Chainho, Maria Ana Dionísio, Maria J. Gaudêncio, Cláudia Afonso, Helena Lourenço, M. Leonor Cancela, Narcisa M. Bandarra, Paulo J. Gavaia

**Affiliations:** ^1^Faculty of Biomedical Sciences and Medicine (FCBM), University of Algarve, Faro, Portugal; ^2^Centre of Marine Sciences, University of Algarve, Faro, Portugal; ^3^Division of Aquaculture, Upgrading and Bioprospection, Portuguese Institute for the Sea and Atmosphere (IPMA), Algés, Portugal; ^4^Interdisciplinary Centre of Marine and Environmental Research (CIIMAR/CIMAR), University of Porto, Porto, Portugal; ^5^Division of Environmental Oceanography, Portuguese Institute for the Sea and Atmosphere, Algés, Portugal; ^6^Marine and Environmental Sciences Centre (MARE), NOVA University of Lisbon, Lisbon, Portugal; ^7^Algarve Biomedical Center (ABC), University of Algarve, Faro, Portugal; ^8^Centre for BioMedical Research (CBMR), University of Algarve, Faro, Portugal

**Keywords:** osteoporosis, natural bioactives, polyphenols, sea cucumbers, tunicates, anti-inflammatory, antioxidants, osteogenic

## Abstract

Bone metabolic disorders such as osteoporosis are characterized by the loss of mineral from the bone tissue leading to its structural weakening and increased susceptibility to fractures. A growing body of evidence suggests that inflammation and oxidative stress play an important role in the pathophysiological processes involved in the rise of these conditions. As the currently available therapeutic strategies are often characterized by toxic effects associated with their long-term use, natural antioxidants and anti-inflammatory compounds such as polyphenols promise to be a valuable alternative for the prevention and treatment of these disorders. In this scope, the marine environment is becoming an important source of bioactive compounds with potential pharmacological applications. Here, we explored the bioactive potential of three species of holothurians (Echinodermata) and four species of tunicates (Chordata) as sources of antioxidant and anti-inflammatory compounds with a particular focus on polyphenolic substances. Hydroethanolic and aqueous extracts were obtained from animals’ biomass and screened for their content of polyphenols and their antioxidant and anti-inflammatory properties. Hydroethanolic fractions of three species of tunicates displayed high polyphenolic content associated with strong antioxidant potential and anti-inflammatory activity. Extracts were thereafter tested for their capacity to promote bone formation and mineralization by applying an assay that uses the developing operculum of zebrafish (*Danio rerio*) to assess the osteogenic activity of compounds. The same three hydroethanolic fractions from tunicates were characterized by a strong *in vivo* osteogenic activity, which positively correlated with their anti-inflammatory potential as measured by COX-2 inhibition. This study highlights the therapeutic potential of polyphenol-rich hydroethanolic extracts obtained from three species of tunicates as a substrate for the development of novel drugs for the treatment of bone disorders correlated to oxidative stress and inflammatory processes.

## Introduction

Non-communicable diseases, also known as chronic diseases, are the utmost burden of the global health systems being by far the major cause of morbidity and deaths (1). Metabolic bone disorders characterized by reduced bone mineral density (BMD), i.e., osteoporosis and osteopenia, are by far the most common diseases related to bone, with a global incidence of 40% in people over the age of 50 (2, 3). The etiology of osteoporosis has been extensively studied over the past decades and a full picture of the underlying molecular mechanisms is being drawn. As such, a growing body of evidence has stressed the importance of inflammation and oxidative stress. The most common type of osteoporosis, for instance, type 1 or postmenopausal osteoporosis, is considered to have pathophysiological roots in the dysregulation of inflammatory processes prompted by the age-related decrease of estrogen levels (4–6).

Nevertheless, current therapeutic approaches for the treatment of osteoporosis do not target to re-equilibrate ROS unbalance nor resolve chronic inflammation. Instead, they rely on anti-resorptive drugs that target osteoclasts-induced bone resorption, or on bone anabolic drugs, which act by pharmacologically increasing osteoblasts-assisted mineral deposition. Despite being successfully implemented in osteoporotic patients, these therapies are still characterized by long-term use-associated side effects and do not lend themselves to the treatment of lifelong chronic conditions like the case of osteoporosis (7).

In this context, natural compounds with antioxidant and anti-inflammatory properties may represent alternative tools for the prevention and treatment of osteoporosis and other chronic diseases (8, 9). In this regard, polyphenols such as flavonoids, and phenolic acids are among the better known naturally occurring antioxidant and anti-inflammatories and have raised the interest of the pharmaceutical industry due to their therapeutic potential for the treatment of chronic disorders (10–12).

In the last decades, the marine environment has emerged as an interesting opportunity to discover new bioactives, and the wide metabolic diversity characterizing marine organisms has drawn the attention of the pharmaceutical industry (13–15). Different classes of marine organisms yielded the discovery of bioactive compounds, with marine invertebrates being the most prolific group (16).

Among these, sea cucumbers, a group of echinoderms belonging to the Class Holothuroidea, are particularly promising. They have long been used in traditional medicine by the communities of Eastern Asia, believed to have health-beneficial properties, and have an appreciable market all around the world (17, 18). Sea cucumbers were previously demonstrated to be able to synthesize, among others, anti-inflammatory and antioxidant compounds (19, 20).

Ascidians, tunicates belonging to the Class Ascidiacea, are another group of marine invertebrates believed to have bioactive potential. Overall, more than a thousand secondary metabolites have been isolated from this group of organisms and several were reported to have biological activity, including antioxidant (21, 22).

Although these two groups of marine animals have been demonstrated to hold great pharmacological potential, a relative scarcity of literature explored their capacity to promote bone formation and mineralization. In the present work, we evaluated the bioactivity of extracts produced from 3 species of sea cucumbers belonging to the Genus *Holothuria – Holothuria (Roweothuria) arguinensis* (Koehler and Vaney, 1906), *H. (Panningothuria) forskali* (Delle Chiaje, 1823), and *H. (Holothuria) mammata* (Grube, 1840), and from 4 species of ascidians belonging to the Families Styelidae – *Styela plicata* (Lesueur, 1823), *Botrylloides diegensis* (Ritter and Forsyth, 1917); Polyclinidae – *Aplidium* sp.; and Cionidae – *Ciona robusta* (Hoshino and Tokioka, 1967) collected from the western coast of Portugal. We extracted the water- and ethanol-soluble phases and explored their antioxidant potential, focusing on their content of polyphenolic compounds, and their anti-inflammatory capacity. Because of the high content of polyphenols and *in vitro* biological activities observed, the extracts were also tested for their capacity to induce bone formation and mineralization *in vivo* by using a screening system based on zebrafish [*Danio rerio*, Hamilton (1882)] larvae, which was previously developed to assess the bone anabolic and pro-osteogenic activity of molecules and extracts, as validated by Tarasco et al. (23).

## Materials and Methods

### Animal Collection and Identification

#### Sea Cucumbers

Fresh samples of sea cucumbers were captured by scuba diving in the coastal zone between Sesimbra and Sado Estuary (Setúbal, Portugal; 38°25′23.50″N; 9°0′45.06″W), from January to July 2019. A total of 208 animals were captured, *Holothuria* (*Roweothuria*) *arguinensis* (*n* = 62), *H.* (*Panningothuria*) *forskali* (*n* = 64) and *H*. (*Holothuria*) *mammata* (*n* = 82). After dissection and removal of internal organs and celomic fluid, specimens were cleaned under running water, minced, and stored at –80°C until analysis.

#### Tunicates

Samples of 4 different tunicates species—*Styela plicata*, *Aplidium* sp., *Botrylloides diegensis*, and C*iona robusta*—were collected by hand as epibionts of mussels cultured in the mussel aquaculture rafts of the Albufeira lagoon. The Albufeira lagoon is a semi-enclosed lagoon located 20 km south of Lisbon on a mesotidal area with a NE-SW orientation to the coast. The mussel rafts are located in the main water bodies of Lagoa Grande with an average depth of 4–5 m.

#### Phylogenetic Identification

Specimens were identified to the lowest possible taxonomic level through integrative taxonomy, combining morphological, and genetic approaches, when necessary. For the morphological approach, a stereomicroscope and identification keys were used. For the genetic approach, a small piece of tissue of each organism was used to extract total DNA using the E.Z.N.A Mollusc DNA Kit (Omega Bio-Tek, Norcross, United States), following the manufacturer’s instructions. An iCycler (Bio-Rad, Hercules, United States) thermal cycler was used to amplify the fragment of the mitochondrial gene cytochrome c oxidase subunit I gene (COI-5P), using a pre-made PCR mix (Invitrogen, Waltham, United States). Each PCR reaction contained 2.5 μL of 10× Taq polymerase buffer, 0.75 μL of 50 mM MgCl2, 0.5 μL of 10 mM dNTP mixture, 0.1 μL of 5 U/μL of Taq DNA polymerase, 1.5 μL (10 μm) of each primer (LoboF1 5′-KBTCHACAAAYCAYAARGAYATHGG-3′ and LoboR1 5′-TAAACYTCWGGRTGWCCRAARAAYCA-3′) (24), 1.25 μL of 1% W-1, 4 μL of DNA template and sterile Milli-Q water to make up a total volume of 25 μL. The conditions of the PCR thermal cycling were as follows: (1) 5 min at 94°C; (2) 5 cycles: 30 s at 94°C, 90 s at 45°C, 60 s at 72°C; (3) 45 cycles: 30 s at 94°C, 90 s at 54°C, 60 s at 72°C; (4) 5 min at 72°C. The amplified PCR products were purified using magnetic beads and subsequently sequenced bidirectionally with the BigDye Terminator 3 kit on an ABI 3730XL DNA analyzer (Applied Biosystems, Waltham, United States) by StabVida. Trace files obtained were carefully analyzed and sequences were aligned using MEGA version X. GenBank BLASTn search (25) and BOLD Identification System tool (BOLD-IDS) (26), were used for matching sequences. The specimens and sequence data obtained in this study is compiled in the Barcode of Life Data Systems project titled “SCUTU—Properties of extracts from sea cucumbers and tunicates.” GenBank accession numbers are presented in Supplementary Table 1.

### Hydroethanolic and Aqueous Extraction

The samples of sea cucumbers and tunicates were freeze-dried in a Heto PowerDry PL3000 Freeze Dryer (Thermo Fisher Scientific, Waltham, United States) for 72 h. Freeze-dried biomass was grounded into a fine powder with a Retsch GM200 ball mill (Retsch GmbH, Haan, Germany). Hydroethanolic (HE) and aqueous (AQ) extracts were prepared through liquid extraction. Biomass powder was weighed and transferred into 50 mL Falcon tubes covered with aluminum foil to prevent photo-oxidative processes to take place and solubilized in distilled water and 96% ethanol-water mixture, respectively. A biomass-solvent ratio of 30 mL of solvent per 1 g of biomass was used. Solutions were homogenized with a UNIDRIVE X1000D Homogenizing System (CAT, Deerfield, United States) at 30,000 rpm for 1 min and then left on an orbital shaker (VWR International, Radnor, United States) overnight (15 h) to extract phenolic compounds. The next day, extracts were centrifuged at 1,000 g for 5 min to allow the insoluble material to precipitate and the supernatant was transferred into new 50 mL Falcon tubes. Centrifugation was repeated 3 times. HE and AQ extracts were aliquoted and a half was immediately used for polyphenol determination and antioxidant activity, while the other half was evaporated and completely dried before testing anti-inflammatory activity and osteogenic activity. Fractions were then evaporated using a rotatory evaporator RV 10 digital (IKA-Werke GmbH & Co., Staufen, Germany), setting a temperature of 40°C for HE extracts and 50°C for AQ extracts, and then stored at –80°C. Before exposure to the fish, extracts were resuspended in their vehicle, ethanol (Merck, Darmstadt, Germany) and Milli-Q water (pH 7.4), respectively.

### Total Polyphenols Content

Polyphenols content was determined in the AQ and HE extracts by colorimetry determination with phosphomolybdic-phosphotungstic acid with a protocol adapted from Singleton and Rossi (27). The assay is based on the reactivity of the Folin-Ciocalteu reagent, which consists of a yellow acidic solution containing complex polymeric ions formed from phosphomolybdic and phosphotungstic acids. In an alkaline solution, this reagent oxidizes phenolic compounds and is reduced producing a complex molybdenum-tungsten blue pigment, which can be spectrophotometrically detected by reading the absorbance at λ = 750 nm. The total polyphenols content is calculated relatively to the reactivity of gallic acid (3,4,5-trihydroxy benzoic acid), a known phenolic compound, under the same conditions. Folin-Ciocalteu reagent (Merck) was diluted 1:1 in Milli-Q water before utilization. For a volume of 100 μL of HE or AQ extract, 600 μL of Milli-Q water and 150 μL of Folin-Ciocalteu reagent were added to test tubes. The mixture was then vortexed and incubated in the dark for 5 min at room temperature. Subsequently, 750 μL of 2% sodium carbonate (Na_2_CO_3_) prepared in water were added to each test tube. Mixtures were then vortexed and incubated in the dark for 90 min at room temperature. Each reaction was performed in triplicate for each extract. Absorbance was then detected at 750 nm using a UV-Vis spectrophotometer Helios Alpha-model (Unicam, Cambridge, United Kingdom). The calibration curve was calculated by using standard solutions of gallic acid (Merck) —0.025, 0.05, 0.1, 0.2, 0.3 mg/mL— obtained by serial dilutions from a stock solution (1 mg/mL) prepared in ethanol for both AQ and HE extracts. Total polyphenols content was assessed in triplicates and calculated from the gallic acid calibration curve as milligrams of gallic acid-equivalent (GAE) polyphenols per gram of dry biomass (mg GAE/100 g dw).

### Biological Activities

#### Antioxidant Activity

Antioxidant potential was assessed for the HE and AQ extracts from sea cucumbers and tunicates. Free radical scavenging capacity was assessed with two assays based on a single electron transfer reaction (FRAP and ABTS^+^ methods), and one assay based on a hydrogen atom transfer reaction (DPPH method), which are considered complementary methods for the evaluation of antioxidant capacity (28, 29). Antioxidant activities were analyzed in triplicate for all the tests performed.

##### Ferric Reducing Antioxidant Power Assay

The ferric reducing antioxidant power (FRAP) was evaluated for HE and AQ extracts with a protocol based on the method developed by Benzie and Strain (30) and adapted by Martins et al. (31). The test is based on a redox reaction occurring between the substrate (electron donor) and Fe^3+^ ions (electron acceptor), producing Fe2^+^ ions. This reduction is monitored spectrophotometrically by the change in the color of the solution of Fe^3+^ with TPTZ [2,4,6-tris(2-pyridyl)-s-triazine], which turns blue and absorbs electromagnetic radiation at λ = 595 nm. The ferric reducing antioxidant power of the extracts is calculated as related to the absorbance of a Fe^2+^ standard solution of iron sulfate (FeSO_4_), tested in parallel. The FRAP reagent was prepared by mixing acetate buffer (0.3 M), TPTZ (10 mM) (Alfa-Aesar, Cityward Hill, United States), and FeCl_3_⋅6H_2_O (20 mM) (Merck) in the ratio 10:1:1. For a volume of 100 μL of HE or AQ extract, 3.0 mL of the FRAP reagent was added to test tubes and the mixture was then incubated in the dark for 30 min at 37°C in a water bath. The absorbance of the samples was read in comparison to the blank (prepared by adding the FRAP reagent to 96% ethanol and water for AQ and HE extracts, respectively) at a wavelength of 595 nm, using a UV-Vis spectrophotometer Helios Alpha-model (Unicam). The calibration curve was prepared using standard solutions of FeSO_4_ (0.25, 0.5, 1, 1.5, 2 mM) obtained by serial dilution from a stock solution (2 mM). All measurements were conducted in triplicate and the results were expressed as micromoles of equivalents of iron sulfate per g of dry biomass (μmol eq FeSO_4_/g dw).

##### ABTS^+^ Assay

The antiradical capacity of HE and AQ extracts was evaluated by following their effect on the stable free cation radical ABTS^+•^ [2,2′-azino-bis (3-ethylbenzothiazoline-6-sulfonic acid)], following the protocol used by Re et al. (32). The assay is based on the scavenging capacity of antioxidants against ABTS^+•^ radical cation, a blue/green chromophore, which has multiple absorbance maxima at 645 nm, 734 nm, and 815 nm. ABTS^+•^ is firstly produced by the reaction between ABTS and potassium persulfate (K_2_S_2_O_8_). The addition of antioxidants reduces it to ABTS resulting in the decolorization of the solution which is monitored via reading the reduction of the absorbance at λ = 734 nm. The scavenging capacity of the ABTS^+•^ radical cation is determined as a function of concentration and time and calculated relative to the reactivity of Trolox (6-hydroxy-2,5,7,8-tetramethychroman-2-carboxylic acid) as antioxidant standard, under the same conditions. ABTS, [2,2′-azino-bis (3-ethylbenzothiazoline-6-sulfonic acid)] diammonium salt, and potassium persulfate (K_2_S_2_O_8_) were obtained from Merck. ABTS^+•^ solution (7 mM) was prepared by dissolving 10 mg of ABTS into a potassium persulfate solution (2.45 mM) prepared in Milli-Q water and incubating the mixture in the dark for 16 h at room temperature. Because ABTS and potassium persulfate reacts stoichiometrically at a ratio of 1:0.5, this results in incomplete oxidation of the ABTS and production of ABTS^+•^. After the incubation, the ABTS^+•^ solution was diluted by adding 5 mM phosphate-buffered saline (PBS), pH 7.4 until obtaining a final absorbance of 0.7 ± 0.02 at 734 nm. For a volume of 20 μL of HE or AQ extract, 2.0 mL of ABTS^+•^ solution was added to test tubes. The mixture was then vortexed and incubated in the dark for 6 min at 37°C in a water bath. Absorbance was detected again at 734 nm using a UV-Vis spectrophotometer Helios Alpha-model (Unicam). The calibration curve was calculated by using standard solutions of Trolox (Merck) (0, 100, 250, 500, 1,000, and 2,000 μM) obtained by serial dilution from a stock Trolox solution (3 mM) prepared in 96% ethanol for AQ and HE. ABTS^+•^ radical scavenging capacity was calculated as micromoles of equivalents of Trolox per 100 g of dry biomass (μmol eq TROLOX/100 g dw).

##### DPPH Assay

Radical scavenging activity of AQ and HE extracts against the stable radical DPPH^•^ (2,2-diphenyl-2-picrylhydrazyl hydrate) was determined spectrophotometrically by following a protocol adapted from Miliauskas et al. (33). When DPPH^•^ reacts with an antioxidant compound able to donate a hydrogen proton, it is reduced to DPPH-H, and the color change which results can be detected by measuring the reduction of absorbance at λ = 517 nm. The scavenging capacity against the stable DPPH^•^ radical is determined relative to the reactivity of ascorbic acid as an antioxidant standard, under the same conditions. DPPH solution (0.15 mM) was prepared by dissolving 11.8 mg of DPPH reagent (2,2-diphenyl-1-picrylhydrazyl, 95%, Alfa-Aesar) into 200 mL of methanol in a volumetric flask. For 1 mL of HE or AQ extract, 2.0 mL of the DPPH solution was added to test tubes. Solutions were vortexed and incubated for 30 min in the dark at room temperature. Reduction in the absorbance at 527 nm was detected with a UV-Vis spectrophotometer Helios Alpha-model (Unicam). The absorption of a blank sample containing the same amount of DPPH^•^ solution and ethanol 96% or water was measured. The experiment was carried out in triplicate for each extract and the controls (water and 96% ethanol for AQ and HE extracts, respectively). A calibration curve with ascorbic acid (Merck) was drawn by reading absorbance (517 nm) of standard concentrations of ascorbic acid (5, 10, 15, and 20 mg/L) obtained by serial dilution from the higher concentration (20 mg/L) and a calibration curve equation was calculated for both HE and AQ extracts. Radical scavenging capacity was calculated as milligrams of equivalents of ascorbic acid per 100 g of dry biomass (mg eq AA/100 g dw).

### Anti-Inflammatory Activity

Anti-inflammatory activity was evaluated by the cyclooxygenase-2 (COX-2) inhibitory assay, an established biomarker of inflammation processes and target of common non-steroidal anti-inflammatory drugs (NSAID) (34). HE and AQ extracts were subjected to heat treatment (80°C during 1 h) and centrifuged (3,000 g at 4°C for 10 min). The supernatant was collected and the solvent was evaporated using a vacuum rotary evaporator with the water bath temperature at 65°C. The residue was directly dissolved in 100% dimethyl sulfoxide (DMSO, Merck) to prepare a stock solution with a concentration of 10 mg⋅mL^−1^. Extracts were tested at 1 mg⋅mL^−1^ using a commercial COX-2 inhibitory screening assay kit, Cayman test kit-560131 (Cayman Chemical Company, Ann Arbor, United States). A volume of 10 μL of each of the tested extracts or DMSO (blank) was used. Results were expressed as a percentage of inhibition of COX-2. Anti-inflammatory activity was analyzed in quadruplicate.

### Acute Toxicity and Osteogenic Activity

**FIGURE 1:**
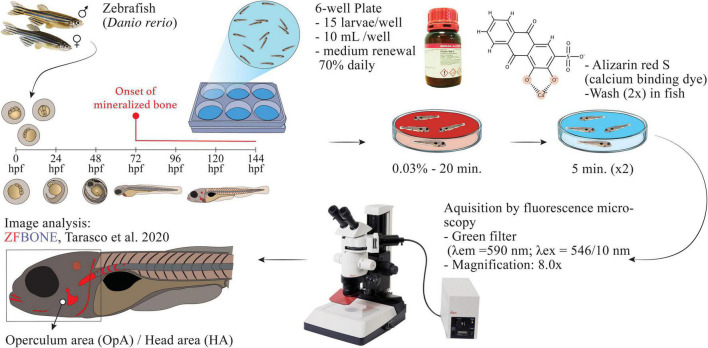
Scheme of the protocol used for the evaluation of the osteogenic activity.

Adult zebrafish (AB wild-type strain) were crossed by using an in-house breeding program. Fertilized eggs were transferred into plastic 1 L tanks with static water conditions and maintained until hatching, at 3 days post-fertilization (dpf), with the following conditions: temperature 28 ± 0.1°C, pH 7.5 ± 0.1, conductivity 700 ± 50 μS, NH_3_ and NO_2_ lower than 0.1 mg/L, NO_3_ at 5 mg/L and a photoperiod of 14:10 h light-dark. Fish water was prepared by adding a salt mixture (Instant Ocean, Blacksburg, United States) and sodium bicarbonate to reverse osmosis treated water in order to maintain stable pH and conductivity. The osteogenic activity was evaluated for HE and AQ extracts as described by Tarasco et al. (23). The process is schematized in Figure 1. Briefly, at 3 dpf, hatched embryos (*n* = 15) were transferred into 6 well-plates and treated with each extract and their vehicle as the control group (ethanol 0.1% for HE and water for AQ extracts). Calcitriol (1α,25-dihydroxy vitamin D_3_, Sigma-Aldrich, St. Louis, United States), was used as a positive control at a concentration of 10 pg/mL in ethanol. Extracts were tested in repetitive experiments at different concentrations and developmental toxicity was evaluated in order to calculate the maximum non-lethal dose (MNLD) over a 72 h period. All extracts were initially solubilized in either ethanol or water at the higher concentration possible. For all extracts, the upper limit of solubility was 200 μg/mL. Toxicity was tracked daily by monitoring the mortality induced in each condition. Larvae were considered not viable when one of the following observations occurred: all or part of larval body degraded, severe growth retardation, a developmental anomaly with severity non-consistent with larval survival. Whenever the extracts resulted in toxicity, the concentration was reduced to 100 μg/mL and the experiment was repeated. At 6 dpf, larvae were sacrificed with a lethal dose of MS-222 (0.6 mM, pH 7.0, Sigma-Aldrich), stained for 20 min at room temperature with 0.03% alizarin red S (AR-S) prepared in Milli-Q water (pH 7.4), and washed twice with Milli-Q water for 5 min. Euthanized larvae were placed in a lateral position on top of a 2.5% agarose gel plate and imaged using a Leica MZ10F fluorescence stereomicroscope (Leica, Wetzlar, Germany) equipped with a green fluorescence filter (λex = 546/10 nm), a barrier filter (λem = 590 nm), and a DFC7000T camera (Leica). Images were acquired using the following parameters: exposure time 300 ms, gamma 1.00, image format 1,920 × 1,440 pixels, binning 1 × 1. Fluorescence images were analyzed using ImageJ software version 2.0.0-rc-69/1.52p. For morphometric analysis, the images were processed by using the toolbox available within the “ZFBONE” macro toolset for Fiji (35). The area of the operculum and the area of the head were digitally measured with the support of an Intous M drawing tablet (Wacom, Ōtone, Japan). The ratio of the operculum area on the head area was calculated as the final value as the area of the head is proven to be the most accurate morphological parameter to normalize inter-specimen size variations (23).

### Statistical Analysis

For all the experiments, normality was tested with a D’Agostino-Pearson omnibus normality test or with an Anderson-Darling test (*p* < 0.05). Homoscedasticity was tested through the Brown-Forsythe test (*p* < 0.05). When the distribution of the data of all the experimental groups resulted normal and homogeneous, statistical differences between the control and the extracts were tested with a one-way ANOVA followed by Dunnett’s multiple comparison test (*p* < 0.05). If the distribution of the data of any of the experimental conditions resulted non-normal or non-homogeneous, statistical differences between the control and the extracts were tested with a non-parametric test followed by Dunn’s multiple comparison test (*p* < 0.05). Statistical analyses were performed using Prism version 8.00 (GraphPad^®^ Software, Inc. La Jolla, United States).

## Results

### Total Polyphenols Content

Tunicate extracts displayed a high content of polyphenolic compounds, with higher values for the HE fractions. The extracts with the highest content were the ones obtained by HE fractions of *Botrylloides diegensis*, 669 ± 11 mg GAE/100 g dw, *Aplidium* sp., 365 ± 21 mg GAE/100 g dw and *Ciona robusta*, 206 ± 14 mg GAE/100 g dw (Table 1). Polyphenol contents from the extracts of holothurians were generally lower than ascidians, never exceeding 100 mg GAE/100 g dw, and were higher in the AQ extraction compared to the HE (Table 1).

**TABLE 1:** Polyphenol content (mg GAE/100 g dw) in aqueous and ethanolic extracts of the studied tunicates and sea cucumbers.

	Polyphenol content (mg GAE/100 g dw)	
	Extract	
Species	Aqueous (*n* = 3)	Ethanolic (*n* = 3)
*Styela plicata*	31 ± 6^aA^	40 ± 7^bA^
*Aplidium* sp.	260 ± 13^dA^	365 ± 21^dB^
*Botrylloides diegensis*	428 ± 10^eA^	669 ± 11^eB^
*Ciona robusta*	103 ± 8^cA^	206 ± 14^cB^
*H. (Roweothuria) arguinensis*	84 ± 4^bcA^	9 ± 5^abB^
*H*. (Holothuria) *mammata*	79 ± 1^bA^	21 ± 17^abB^
*H. (Panningothuria) forskali*	48 ± 2^aA^	nd^aB^

*Values are presented as average ± standard deviation. For all groups, n = 3. Nd, not detected. Different lowercase letters within a column correspond to statistical differences (p < 0.05) between different species and same extract type (ethanolic or aqueous, respectively). Different uppercase letters within a row correspond to statistical differences (p < 0.05) between aqueous and ethanolic extracts from the same species.*

### Antioxidant Activity

The antioxidant activity was measured by the use of three alternative methodologies (ABTS, FRAP, and DPPH) in the extracts from the studied tunicates and sea cucumbers (Table 2). Three species of tunicates—*Aplidium* sp., *Botrylloides diegensis*, and *Ciona robusta*—displayed the highest antioxidant activities with consistent results across the different methodologies used. When measured by DPPH and FRAP assays, the HE extracts were the ones displaying higher antioxidant activity. However, when tested with ABTS assay, the AQ extract also displayed high antioxidant activity, surpassing the ones of the HE extracts. Moderate antioxidant activity was reported also for the AQ extracts from *H. arguinensis* and *H. mammata* when assessed by ABTS assay, but always lower compared to tunicates.

**TABLE 2:** Antioxidant activity as measured by ABTS (μmol eq TROLOX/100 g dw), FRAP (μmol eq FeSO4/g dw), and DPPH (mg eq AA/100 g dw) in aqueous and ethanolic extracts of the studied tunicates and sea cucumbers.

	ABTS (μ mol eq TROLOX/100 g dw)		FRAP (μ mol eq FeSO_4_/g dw)		DPPH (mg eq AA/100 g dw)	
	Extract		Extract		Extract	
Species	Aqueous (*n* = 3)	Ethanolic (*n* = 3)	Aqueous (*n* = 3)	Ethanolic (*n* = 3)	Aqueous (*n* = 3)	Ethanolic (*n* = 3)
*Styela plicata*	1,126 ± 81^aA^	552 ± 60^bB^	2.9 ± 0.5^aA^	5.5 ± 0.1^bB^	6.9 ± 10.9^abA^	nd^aB^
*Aplidium* sp.	6,206 ± 180^fA^	5,020 ± 20^dB^	34.3 ± 0.4^eA^	34.2 ± 0.1^dA^	nd^aA^	102.0 ± 1.6^bB^
*Botrylloides diegensis*	6,735 ± 11^gA^	5,886 ± 83^eB^	54.6 ± 0.4^fA^	56.5 ± 0.0^eB^	16.7 ± 4.1^bcA^	179.0 ± 1.7^cB^
*Ciona robusta*	4,271 ± 109^eA^	3,027 ± 56^cB^	19.7 ± 0.6^dA^	15.6 ± 0.6^cB^	151.7 ± 4.4^eA^	162.7 ± 2.5^cB^
*H. arguinensis*	2,234 ± 136^cA^	166 ± 35^aB^	7.0 ± 0.2^bA^	2.0 ± 0.1^aB^	nd^aA^	13.5 ± 12.7^aB^
*H. mammata*	3,089 ± 158^dA^	33 ± 57^aB^	6.2 ± 0.0^bA^	1.5 ± 0.2^aB^	31.8 ± 6.2^cA^	5.7 ± 9.8^aB^
*H. forskali*	1,883 ± 81^bA^	nd^aB^	8.9 ± 0.5^cA^	2.1 ± 0.6^aB^	106.1 ± 4.7^dA^	nd^aB^

*Values are presented as average ± standard deviation. nd, not detected. For all groups, n = 3. Different lowercase letters within a column correspond to statistical differences (p < 0.05) between different species and same extract type (ethanolic or aqueous, respectively). For each antioxidant methodology, different uppercase letters within a row correspond to statistical differences (p < 0.05) between aqueous and ethanolic extracts from the same species.*

### Anti-inflammatory Activity

The anti-inflammatory activity was measured by the percentage of inhibition of COX-2 (Table 3). First, HE extracts generally showed higher anti-inflammatory activity than the AQ extracts. HE fractions from sea cucumbers exhibited relatively low values of COX-2 inhibition ranging from 16.4 to 41.8%, while the AQ extracts did not present any detectable activity. AQ extracts from tunicates presented a moderate anti-inflammatory activity, ranging from 8.9 ± 3.7% in of *Botrylloides diegensis* to 33.2 ± 5.2% for *Ciona robusta*. However, the HE fractions from the four species of tunicates showed high anti-inflammatory activity, three of which in the 70.2–76.7% interval, while the highest activity was measured for the extracts from *Ciona robusta*, showing 92.2 ± 8.5% inhibition of COX-2 activity.

**TABLE 3:** Anti-inflammatory activity (% inhibition of COX-2) in aqueous and ethanolic extracts of the studied tunicates and sea cucumber species.

	Anti-inflammatory activity (% inhibition COX-2)	
	Extract	
Species	Aqueous (*n* = 4)	Ethanolic (*n* = 4)
*Styela plicata*	19.6 ± 5.8^cA^	76.7 ± 3.4^cB^
*Aplidium* sp.	27.8 ± 2.5^dA^	70.2 ± 6.3^cB^
*Botrylloides diegensis*	8.9 ± 3.7^bA^	75.9 ± 3.0^cB^
*Ciona robusta*	33.2 ± 5.2^dA^	92.2 ± 8.5^dB^
*H. (Roweothuria) arguinensis*	nd^aA^	40.0 ± 7.6^bB^
*H. (Holothuria) mammata*	nd^aA^	41.8 ± 4.9^bB^
*H. (Panningothuria) forskali*	nd^aA^	16.4 ± 9.7^aB^

*Values are presented as average ± standard deviation. nd, not detected. For all groups, n = 4. Different lowercase letters within a column correspond to statistical differences (p < 0.05) between different species within same type of extract. Different uppercase letters within a row correspond to statistical differences (p < 0.05) between aqueous and ethanolic extracts from the same species.*

### Developmental Toxicity and Establishment of Maximum Non-lethal Dose

Acute developmental toxicity was evaluated in zebrafish larvae and the Maximum Non-Lethal Dose (MNLD) of each extract was determined during the operculum assay and results are summarized in Table 4. None of the extracts resulted in toxicity at the higher concentration tested (200 μg/mL) but the HE extracts of *H. forskali*, which induced mortality. Because of this observation, all HE extracts were also tested at 100 μg/mL. Control groups—Calcitriol (10 pg/mL), water, and ethanol 0.1% (v/v) never resulted in mortality in any of the assays performed.

**TABLE 4:** Acute developmental toxicity of the AQ and HE extracts from sea cucumbers and tunicates assessed in zebrafish larvae.

Species	Extract	Concentration (μ g/mL)	S_72 h_	Concentration (μ g/mL)	S_72 h_
*Styela plicata*	AQ	200*	15/15	–	–
*Styela plicata*	HE	200*	15/15	100	15/15
*Aplidium* sp.	AQ	200*	15/15	–	–
*Aplidium* sp.	HE	200*	15/15	100	15/15
*Ciona robusta*	AQ	200*	15/15	–	–
*Ciona robusta*	HE	200*	15/15	100	15/15
*Botrylloides diegensis*	AQ	200*	15/15	–	–
*Botrylloides diegensis*	HE	200*	15/15	100	15/15
*H. (Panningothuria) forskali*	AQ	200	15/15	–	–
*H. (Panningothuria) forskali*	HE	200	11/15	100*	15/15
*H. (Roweothuria) arguinensis*	AQ	200*	15/15	–	–
*H. (Roweothuria) arguinensis*	HE	200*	15/15	100	15/15
*H. (Holothuria) mammata*	AQ	200*	15/15	–	–
*H. (Holothuria) mammata*	HE	200*	15/15	100	15/15

Two concentrations have been tested for each extract. Final survival after 72 h of exposure (S_72 h_) was calculated. Depending on the results of the experiment, the concentrations were either increased to a maximum of 200 μg/mL or decreased following a half-logarithmic dilution (3.16, 10, 31.6, 100 μg/mL). Maximum non-lethal dose (*, bold); concentration not tested (–).

### Osteogenic Activity

**FIGURE 2:**
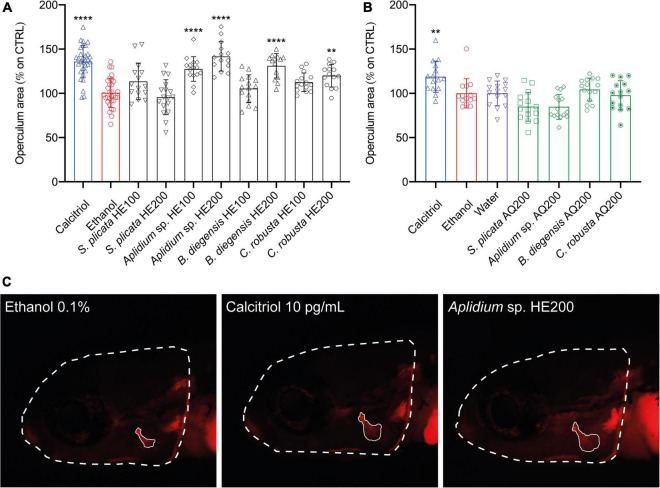
Osteogenic activity of aqueous **(A)** and hydroethanolic **(B)** extracts from four species of tunicates in zebrafish larvae. Results are displayed as corrected operculum area (operculum area/head ratio) expressed as percentage of increase over the control. Representative image **(C)** of a fish treated with the negative control (ethanol), the positive control (Calcitriol 10 pg/mL) and the most powerful osteogenic extracts among tunicates (*Aplidium* sp. HE200). Statistical differences among the means were tested through one-way ANOVA followed by Dunnett’s multiple comparison test (*p* < 0.05) or, whenever normality and homoscedasticity weren’t met, through a non-parametric test followed by Dunn’s multiple comparison test (*p* < 0.05). *P*-values are indicated as follow: 0.0021. (**), < 0.0001 (****). HE, hydroethanolic extracts, AQ, aqueous extracts, 100–100 μg/mL, 200–200 μg/mL.

To explore the pro-osteogenic potential of the extracts studied, the effect of HE and AQ extracts on bone formation during zebrafish development was assessed using the operculum assay method (23). None of the extracts significantly affected the area of the head, indicating that there was not a significant variation in growth among treatments (Supplementary Figure 1). Overall, hydroethanolic extraction was able to isolate compounds with pro-osteogenic bioactivity. In particular, HE fractions from three tunicates species (*Aplidium* sp., *Botrylloides diegensis*, and *Ciona robusta*) were characterized by high osteogenic activity, inducing an increase in the mineralized area of the opercular bone by 41.7 ± 16.6%, 31.1 ± 13.8%, and ± 20.0 12.7%, respectively, all at the concentration of 200 μg/mL (Figure 2C). Statistical differences between the two concentrations tested for the HE extracts were reported for *Aplidium* sp. (*P* = 0.0186) and *Ciona robusta* (*P* < 0.0001) indicating that a dose-dependent effect was present, but not for *Botrylloides diegensis* (*P* = 0.0975).

**FIGURE 3:**
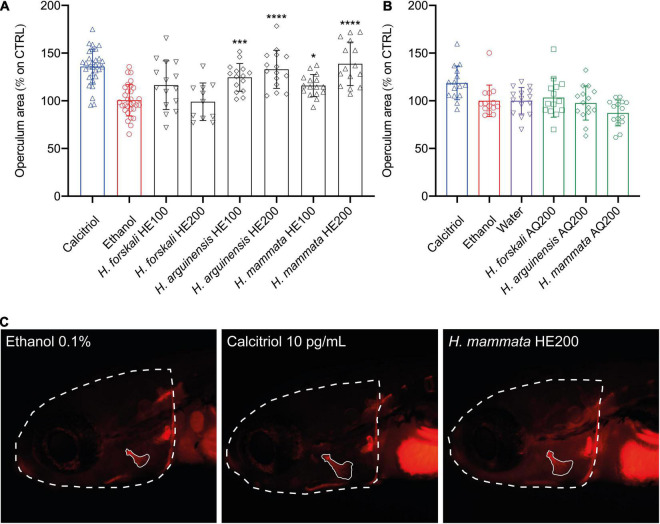
Osteogenic activity of aqueous **(A)** and hydroethanolic **(B)** extracts from three species of sea cucumbers in zebrafish larvae. Results are displayed as corrected operculum area (operculum area/head ratio) expressed as percentage of increase over the control. Representative image **(C)** of a fish treated with the negative control (ethanol), the positive control (Calcitriol 10 pg/mL) and the most powerful osteogenic extracts among holothurians (*H. mammata* HE200). Statistical differences among the means were tested through one-way ANOVA followed by Dunnett’s multiple comparison test (*p* < 0.05) or, whenever normality and homoscedasticity weren’t met, through a non-parametric test followed by Dunn’s multiple comparison test (*p* < 0.05). *P*-values are indicated as follow: 0.0332 (*), 0.0002 (***), < 0.0001 (****). HE, hydroethanolic extracts, AQ, aqueous extracts, 100–100 μg/mL, 200–200 μg/mL.

In contrast, the AQ extracts did not display any evident osteogenic activity at the concentrations tested. Interestingly, also the HE extracts from two species of sea cucumbers, *H*. *arguinensis* and *H*. *mammata*, induced a potent pro-osteogenic effect by increasing the area of the opercular bone in 33.0 ± 19.98%, and 38.8 ± 22.8%, respectively (Figure 3). For holothurians HE extracts, when differences between 200 vs. 100 μg/mL were tested through Student’s *t*-test (*P* < 0.05), there were no statistical differences for *H. arguinensis* (*P* = 0.1942), while these were present for *H. mammata* (*P* = 0.0014).

## Discussion

Given the compelling need for novel therapies for the treatment of bone disorders associated with mineral loss, and considering the rising interest of the pharmaceutical industry toward marine bioactive compounds, we decided to explore the bioactive potential of two somewhat poorly studied groups of marine invertebrates—Sea cucumbers (Holothuroidea) and ascidians (Ascidiacea). Aqueous (AQ) and hydroethanolic (HE) extracts from three species of marine holothurians—*Holothuria arguinensis*, *H. forskali*, and *H. mammata* and 4 species of ascidians: *Styela plicata*, *Aplidium* sp., *Botrylloides diegensis*, and *Ciona robusta* were produced. As the increasing body of evidences suggest that oxidative stress and inflammation are related to the etiology of osteoporosis (4), we screened the extracts for their antioxidant and anti-inflammatory activities by applying several *in vitro* assays. Being polyphenolic compounds one of the most relevant classes of naturally occurring compounds, with recognized antioxidant and anti-inflammatory properties, we determined their content in the extracts.

Concerning the sea cucumbers, previous reports described the presence of compounds with antioxidant activity, including polyphenols, in extracts of sea cucumbers (36–38). A significant presence of phenolic substances was observed in species closely related to the ones studied in the present work including *Holothuria atra* (36), *H. scabra* (37), and one species analyzed in the present work, *H. arguinensis* (38). In the study by Roggatz et al. (38), a cold-water extract of *H. arguinensis* was found to have a polyphenol content of 14.2 mg GAE/100 g dw, while no polyphenols were detected in the ethanolic extract. Similarly, in our study a higher content of polyphenols was found in the AQ extracts compared with the HE. However, the extracts from sea cucumber showed lower content of polyphenolic compounds, when compared with the ones from tunicates. Accordingly, a lower antioxidant and anti-inflammatory potential were also reported for these species. Results from ABTS assay highlighted a moderate antioxidant potential for the AQ extracts of the three holothurians studied, but this observation was not consistent with the antioxidant capacity when tested though FRAP assay, which is based on a similar mechanism used to determine the presence of potential electron donors. On the other side, the DPPH assay, which is used for the determination of compounds with proton (H^•^-) donor potential, did not reveal a high antioxidant activity when compared with the other species studied. The presence of polyphenolic compounds and a moderate antioxidant potential reported for the AQ extracts from holothurians may indicate that these species of sea cucumbers deserve further attention as natural sources of polyphenolic compounds.

Tunicates, on the other side, yielded the most interesting results in terms of all bioactivities analyzed. In literature, very few studies investigated the presence of antioxidant compounds in tunicate species. A radical scavenging potential for hot water extracts of *Styela clava* measured by ABTS assay was previously reported (22). Consistently, we observed antioxidant activity on both AQ and HE extracts for the closely related species *Styela plicata* studied in this work, measured by ABTS, DPPH, and FRAP assays. However, the species showing higher antioxidant potential were three tunicates (*Aplidium* sp., *Botrylloides diegensis*, and *Ciona robusta*). These species presented the highest content of polyphenols, for both aqueous and hydroethanolic extractions, but with the HE extracts yielding higher contents. The same three species were also characterized by the higher antioxidant activities, as measured by different methodologies. In this regard, a positive correlation was found between polyphenol content and antioxidant potential measured with all the three methodologies used, namely, through the DPPH (*R*^2^ = 0.29, *P* = 0.0311), ABTS (*R*^2^ = 0.70, *P* < 0.0001), and FRAP (*R*^2^ = 0.32, *P* = 0.0212), supporting the hypothesis that the antioxidant potential observed for the tunicates HE extracts may be related to their high content in polyphenolic compounds. HE fractions from all the four species of tunicates also presented the highest anti-inflammatory potential among all the extracts. No previous studies investigated the presence of polyphenolic compounds with anti-inflammatory activity in tunicates, but other compounds with anti-inflammatory properties were already described. For instance, it was previously reported the presence of a dermatan sulfate, similar in structure to the mammalian heparin, isolated from *Styela plicata* that was shown to display anti-inflammatory activity (39). Moreover, two new tricyclic thiazine-containing quinolinequinone alkaloids, ascidiathiazones A and B, that were isolated from *Aplidium* sp. collected from the coast of New Zealand, were reported to inhibit the production of superoxide by human neutrophils (40).

Following the promising results obtained with antioxidant and anti-inflammatory activities, we decided to test the extracts for their osteogenic potential. In this scope, zebrafish offers an interesting opportunity to explore the potential of bioactive compounds *in vivo*, presenting several advantages in comparison to commonly used mammalian models. Small size, high fecundity, short life span, and a relatively high genetic homology with humans, are some of the technical advantages zebrafish has to offer that persuaded researchers in the field of drug discovery to implement it as an animal model for pre-clinical trials (41, 42). Moreover, the translucency of the early larval stages has proved to be very useful for microscopy applications in bone biology and other fields, allowing the observation of the skeletal structures following the staining with dyes with calcium-binding affinity (43). As result, several zebrafish-based assays were developed in recent years for the screening of drugs and natural compounds with the capacity to induce bone formation and mineralization (23, 44) and were successfully used for the screening of osteoactive compounds, including the zebrafish operculum assay (45, 46).

For the AQ extracts from holothurians, the antioxidant activity reported was not translated into an appreciable effect in terms of induction on bone formation and mineralization *in vivo*. Conversely, HE fractions from two species of holothurians—*H. mammata* and *H. arguinensis* displayed a pro-osteogenic effect in zebrafish larvae. However, these extracts were characterized by lower polyphenolic content, antioxidant activities, and anti-inflammatory activities, compared with the tunicates, suggesting that non-phenolic compounds in holothurians may be involved in the effect observed. Nevertheless, holothurians have been described to be able to synthesize compounds with pro-osteoblastogenic potential, as observed by increased viability and activity of alkaline phosphatase in a human osteoblastic cell line (47) and in rat bone marrow mesenchymal stem cells (48). These observations highlight the need for further investigating these two species of holothurians as potential sources of osteogenic bioactives.

Concerning the tunicates, while AQ extracts did not induce any osteogenic effect, the HE extracts from three species – *Aplidium* sp., *Botrylloides diegensis*, and *Ciona robusta* – were characterized by an increase in mineralized area of the opercular bone in zebrafish larvae. This increase was in a similar extent to the positive control (Calcitriol), and in the case of *Aplidium* sp. at 200 μg/mL (*Aplidium* sp. HE200), even surpassing the positive control value. These observations indicate that pro-osteogenic compounds were isolated through hydroethanolic extraction from the three tunicates species. It is important to notice that at the stage of zebrafish development used for analysis, neither active osteoclasts nor bone resorption occurs (23, 49), therefore the effect here observed for the extracts is due to pro-osteogenic rather than anti-resorptive mechanisms.

The causes for osteoporosis have been extensively studied including the involvement of oxidative stress and inflammation that are linked to altered bone metabolism and reduction of bone mineral density. The molecular landscape associated with this process was widely reviewed by Weitzmann and Pacifici (4). Estrogens regulate both osteoblastic and osteoclastic cells through the estrogen receptor (ER), and elevated levels of estrogens during young age act in both a bone anabolic and anti-resorptive manner, contributing to the maintenance of the equilibrium between bone formation and bone resorption, paramount for a functional bone remodeling. Adequate estrogen levels prolong osteoblast and osteocyte life-span (50), while reducing it in osteoclasts (8, 51). Furthermore, the depletion in circulating estrogens following menopause leads to an increased thymic function, otherwise inhibited by estrogens, which results in an elevated production of pro-inflammatory cytokines including IL-1, IL-6, IL-7, and TNF-α, and to the overall increase of TNF-producing T cells populations within bone marrow, thymus and peripheral lymphoid organs (4). Activated T lymphocytes release TNF, that has been shown to inhibit the differentiation of osteoblastic cells (52). Additionally, it increases osteoclast differentiation by stimulating the production of RANKL by osteoblasts and bone marrow stromal cells (53), and increase the sensitivity to RANKL signaling by upregulating the expression of RANK receptor in pre-osteoclastic cells (54). The abnormally increased osteoclastogenesis that results from this process leads to an overregulated and then to a slowered yet continuous bone resorption, which is not compensated by an equally increased bone formation (4).

The age-related increase in oxidative stress within the bone tissue contributes to exacerbate the pathophysiology of postmenopausal osteoporosis (55). ROS are known to stimulate the activation of T cells (56), increase osteoclastic differentiation (57) and induce osteoblast apoptosis (58, 59). A sufficient estrogen level is believed to prevent the instauration of oxidative stress in bone either by directly inhibiting peroxidative processes, upregulating the expression of antioxidant enzymes, or by suppressing the production of ROS (60). Oxidative stress has been recently proposed as major factor in the development of postmenopausal osteoporosis (61).

The use of novel approaches such as the application of treatments with natural-derived products that promote bone-anabolic effects, by counteracting the pro-oxidative and pro-inflammatory processes, without inducing undesired side effects, is paramount for the development of next-generation anti-osteoporotic drugs.

Overall, the *in vivo* pro-osteogenic effect combined with the high content of polyphenols, antioxidant, and anti-inflammatory activities of these species led us to formulate the hypothesis that the pro-osteogenic activity observed for the tunicates HE extracts can be due to polyphenolic compounds. Previous reports showed that polyphenols can stimulate osteoblast function by directly interacting with different molecular pathways involved in osteoblast differentiation (62, 63), by attenuating detrimental effects from pro-inflammatory signals (64) and by exerting a protective effect against reactive oxygen species on osteoblastic cells (65). Interestingly, a positive correlation was found between the pro-osteogenic and the anti-inflammatory bioactivities for the extracts from tunicates (*R*^2^ = 0.59, *P* = 0.0266). This may indicate that the presence of an anti-inflammatory activity can be directly involved in the pro-osteogenic effect reported for the extracts.

Due to their richness in polyphenolic compounds, antioxidant and anti-inflammatory activities associated with their high pro-osteogenic potential, we identified potential candidates for the discovery of compounds with anti-osteoporotic potential in three species of tunicates *Aplidium* sp., *Botrylloides diegensis*, and *Ciona robusta*. Nevertheless, further investigation aimed at evaluating the toxicity and safety of the studied extracts in other models must be conducted and the chemical characterization of these extracts will be necessary to identify phenolic compounds responsible for the effect in the animal model. With this aim, a good strategy may involve the application of Liquid Chromatography and Mass Spectrometry (LC-MS). Furthermore, the use of a transcriptomic approach would be suitable to elucidate the mechanisms of action of the extracts, which may be acting through a synergy of different mechanisms. The application of extracts to zebrafish reporter lines for osteoblastic cells already available (66, 67), may help investigate the involvement and dynamics of specific cellular types modulated by the extracts. Moreover, by testing the extracts on juvenile stages, when osteoclasts are active, it will allow to determine potential effects on bone resorption. Subsequently, it will be necessary to validate whether the activity, as here observed in the zebrafish ontogenetic model, could efficiently translate into the rescue of disease phenotypes in osteoporotic models. In this regard, a medaka (*O. latipes*) model of genetically induced osteoporosis (68) was previously developed and it would be suitable for this purpose. The application to mammalian models of osteoporosis will be important to validate the translational applications of these promising set of extracts to mammalians. In this context, ovariectomized rat is a well-established animal model that can be used for the validation of anti-osteoporotic compounds (69, 70). Overall, the present study provides a first validation of the pro-osteogenic activity of extracts rich in polyphenols, antioxidant and anti-inflammatory compounds, highlighting the suitability of the studied species for the discovery of novel compounds with anti-osteoporotic potential.

## Conclusion

In the present work we explored the potential of extracts from three species of sea cucumbers and four species of tunicates as source of bioactive compounds to be used toward the prevention and treatment of chronic bone disorders characterized by mineral loss, such as osteoporosis and osteopenia. A total of 14 aqueous and hydroethanolic extracts were produced and screened for their content of polyphenols and their antioxidant, anti-inflammatory, and osteogenic potentials. Overall, three species of ascidians showed the strongest antioxidant activities associated with high polyphenols content. These polyphenol-rich extracts were also characterized by the strongest anti-inflammatory activity and a high osteogenic activity by promoting bone formation in zebrafish larvae. We hypothesize that the positive effect on bone formation observed for the HE extracts from the tunicates studied can be due to the antioxidant and anti-inflammatory properties of polyphenolic compounds isolated through hydroethanolic extraction. Regarding the extracts from sea cucumbers, these generally showed a lower antioxidant activity, although moderate anti-inflammatory and strong pro-osteogenic activities were yielded by the hydroethanolic extraction. These findings provide an evidence of the presence of bioactive compounds with potential for therapeutic applications in the treatment of metabolic bone disorders, opening the opportunity for further investigation.

## Data Availability Statement

The raw data supporting the conclusions of this article will be made available by the authors, without undue reservation.

## Ethics Statement

The animal study was reviewed and approved by the Direção Geral de Alimentação e Veterinária approval number 012769 from 2021.

## Author Contributions

AC, CC, MC, NB, and PG: conceptualization and investigation. AC, CC, DJ, IF, JL-A, SS, PC, and MD: methodology. CC, MC, NB, PG, and MG: resources. AC and CC: data curation. AC, CC, NB, and PG: writing. AC, CC, NB, PG, JL-A, and SS: review and editing. CC, NB, PG, MC, and MG: supervision. CC, NB, MC, and PG: funding acquisition. All authors contributed to the article and approved the submitted version.

## Conflict of Interest

The authors declare that the research was conducted in the absence of any commercial or financial relationships that could be construed as a potential conflict of interest.

## Publisher’s Note

All claims expressed in this article are solely those of the authors and do not necessarily represent those of their affiliated organizations, or those of the publisher, the editors and the reviewers. Any product that may be evaluated in this article, or claim that may be made by its manufacturer, is not guaranteed or endorsed by the publisher.

## Supplementary Material

Supplementary Figure 1 Effect of the treatment with different extracts on the area of the head of zebrafish larvae for holothurians hydroethanolic **(A)** and aqueous extracts **(B)** and for tunicates hydroethanolic **(C)** and aqueous extracts **(D)** respectively. Statistical differences among the means were tested through One-way ANOVA followed by Dunnett’s multiple comparison test (*p* < 0.05) or, whenever normality and homoscedasticity weren’t met, through a non-parametric test followed by Dunn’s multiple comparison test (*p* < 0.05). HE – hydroethanolic extracts, AQ – aqueous extracts, 100 – 100 μg/mL, 200 – 200 μg/mL.

Supplementary Table 1 GenBank accession numbers for the sequence data obtained for the identification of four species of Tunicates.
