# Supplementary figures and images for: Antioxidant and Anti-inflammatory Extracts From Sea Cucumbers and Tunicates Induce a Pro-osteogenic Effect in Zebrafish Larvae
Source: Front Nutr. 2022 May 9;9:888360. doi: 10.3389/fnut.2022.888360 (PMC9125325; doi:10.3389/fnut.2022.888360)

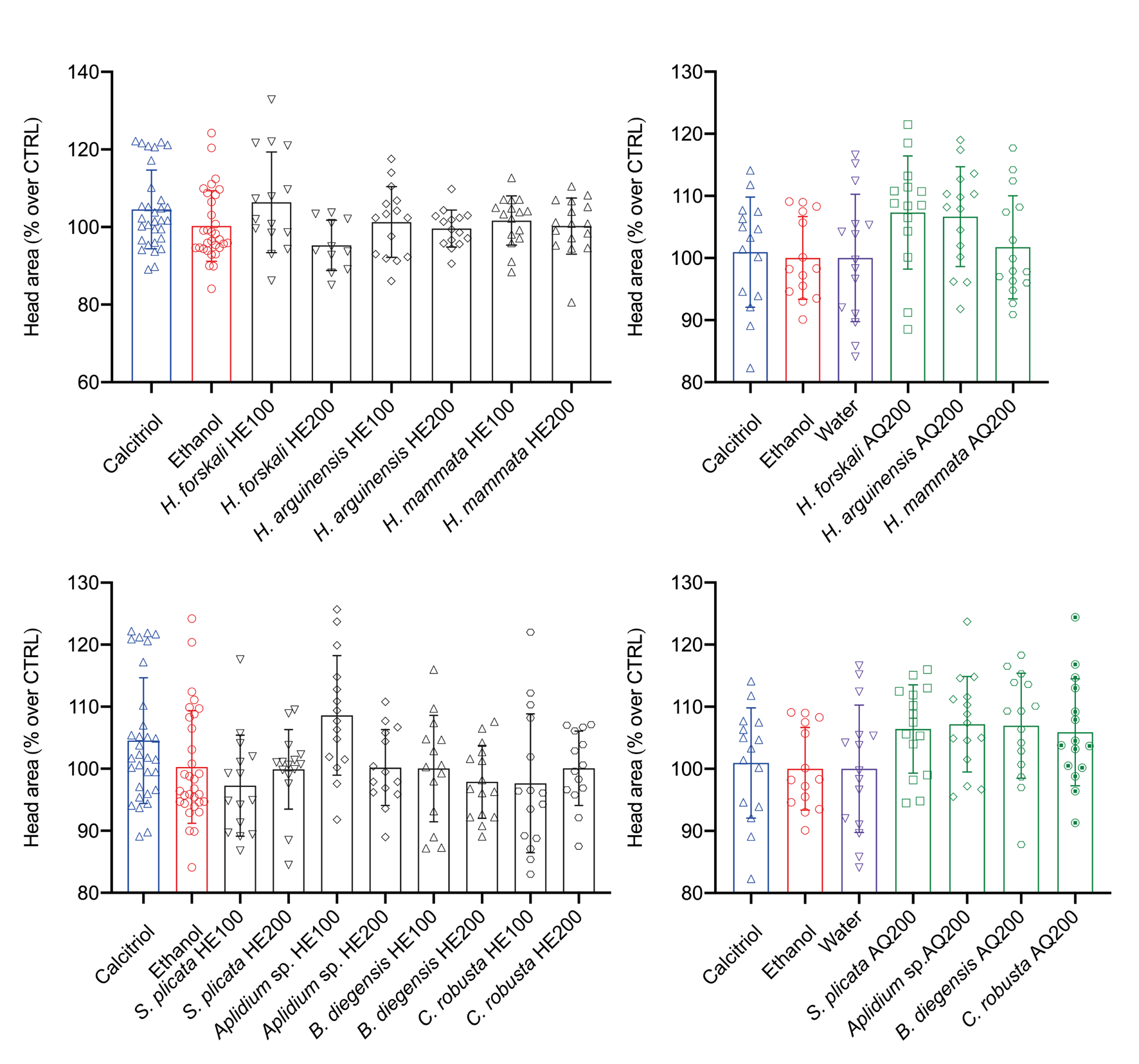

Supplement: Supplementary Figure 1 — Effect of the treatment with different extracts on the area of the head of zebrafish larvae for holothurians hydroethanolic (A) and aqueous extracts (B) and for tunicates hydroethanolic (C) and aqueous extracts (D) respectively. Statistical differences among the means were tested through One-way ANOVA followed by Dunnett’s multiple comparison test (p < 0.05) or, whenever normality and homoscedasticity weren’t met, through a non-parametric test followed by Dunn’s multiple comparison test (p < 0.05). HE – hydroethanolic extracts, AQ – aqueous extracts, 100 – 100 μg/mL, 200 – 200 μg/mL. [file Image_1.TIF]
