# Supplementary material for: Antioxidant and Anti-inflammatory Extracts From Sea Cucumbers and Tunicates Induce a Pro-osteogenic Effect in Zebrafish Larvae
Source: Front Nutr. 2022 May 9;9:888360. doi: 10.3389/fnut.2022.888360 (PMC9125325; doi:10.3389/fnut.2022.888360)
Supplement: Supplementary Table 1 — GenBank accession numbers for the sequence data obtained for the identification of four species of Tunicates. [file Data_Sheet_1.pdf]

SUPPLEMENTARY MATERIAL

Supplementary Table I. GenBank accession numbers for the sequence data obtained for the identification of four species of Tunicates.

Species	BOLDSystems Process ID	GenBank accession number
Aplidium sp.	SCUTU005-22	ON059139
Aplidium sp.	SCUTU006-22	ON059140
Botrylloides diegensis	SCUTU008-22	ON059141
Ciona robusta	SCUTU010-22	ON059142
Ciona robusta	SCUTU009-22	ON059143
Holothuria (Roweothuria) arguinensis	SCUTU001-22	ON059144
Holothuria (Panningothuria) forskali	SCUTU002-22	ON059145
Holothuria (Holothuria) mammata	SCUTU004-22	ON059146
Holothuria (Holothuria) mammata	SCUTU003-22	ON059147
Styela plicata	SCUTU007-22	ON059148

SUPPLEMENTARY FIGURE LEGENDS

Supplementary Figure 1. Effect of the treatment with different extracts on the area of the head of zebrafish larvae for holothurians hydroethanolic (**A**) and aqueous extracts (**B**) and for tunicates hydroethanolic (**C**) and aqueous extracts (**D**) respectively. Statistical differences among the means were tested through One-way ANOVA followed by Dunnett's multiple comparison test ($p < 0.05$) or, whenever normality and homoscedasticity weren't met, through a non-parametric test followed by Dunn's multiple comparison test ($p < 0.05$). HE - hydroethanolic extracts, AQ – aqueous extracts, 100 – 100 µg/mL, 200 – 200 µg/mL.
